# Adipokines and their role in chronic kidney disease

**Published:** 2015-10-27

**Authors:** Majid Khazaei

**Affiliations:** ^1^Neurogenic inflammation Research Center and Department of Physiology, Mashhad University of Medical Sciences, Mashhad, Iran

**Keywords:** Obesity, Adipokines, Kidney

**Implication for health policy/practice/research/medical education:**

Obesity is associated with increase in mortality and morbidity due to renal dysfunction. Adipocyte cells are able to secrete different protein named adipokines. In obese subjects, the production and secretion of adipokines alter and these may affect renal homeostasis. More study in the effect of adipokines in progression of chronic kidney disease can help us to understand the pathophysiology and better management of kidney disease in obese patients.

A recent review paper published in “*Journal of Nephropathology*” discussed the relationship between obesity and renal function (1). Obesity has several related pathologies including cardiovascular and renal disorders. Studies reported that the end stage renal disease in obese patients is increased worldwide during the last 10 years. Both structural (glomerulus) and functional (proximal tubule) changes have been found in obesity (2).

More recently, adipose tissue has been recognized as an active endocrine organ which secretes a variety of cytokines that are called adipokines. Adipokines have several roles in vivo especially in atherosclerosis, inflammation, vascular homeostasis. Leptin, a 16-kDa peptide, is predominantly secreted by adipose tissue which has different role in vivo such as regulation of satiety, energy homeostasis and inflammation. In obese subjects, the plasma leptin level is increased (3). Studies have shown that in patients with chronic kidney disease (CKD), serum leptin is elevated and correlated with serum C-reactive protein (CRP) level (4). The kidney has abundant leptin receptor which expressed on glomerular endothelial cells and mesengial cells. Elevated level of leptin causes basement membrane thickening and glomerular mesengial hypertrophy which leads to tubular apoptosis and structural changes (2,5). Leptin may promote renal fibrosis through signal transduction pathway involving phosphatidyl inositol-3-kinase (5). Leptin also involves in inflammatory process. Administration of leptin to obese animals stimulates T-cell production, renal macrophage infiltration, T-cell survival which may partly explain the hypertrophic changes in the kidney (6,7).

Adiponectin is a 30-kDa plasma protein which is the most abundant adipose tissue protein. It has several physiological roles including insulin-sensitizing effect, cardiovascular protection and anti-inflammation (2,8). Plasma adiponectin level is inversely related to percent body fat. It is associated with higher levels of IL-6 and CRP which involved in renal dysfunction (2,8). Overexpression of adiponectin in rat improved renal function and proteinuria in diabetic nephropathy (9). Hypoadiponectinemia in obese animals causes upregulation of podocyte production of Nox4 and it is suggested the role of oxidative stress in kidney damage (8). It also induces tubular inflammation and accumulation of MCP-1 by decreasing AMPK activation (10). Thus, low plasma adiponectin level can be a marker for progression of kidney damage.

Resistin is 12.5-kDa protein which produced in macrophage and in lower levels by adipocytes. Plasma resistin level is increased in CKD and associated with reduced glomerular filtration rate and inflammation (11). In obese subjects, plasma resistin level are elevated. The role of resistin in obesity is not exactly determined, however, obesity is accompanied with macrophage invasion in the kidney (2) and it seems that it causes endothelial dysfunction which leads to glomerulosclerosis and interstitial fibrosis.

Visfatin is a 52-kDa protein which primarily secreted by adipocyte cells. Obesity is associated with elevated visfatin level. Visfatin increases the production of different inflammatory markers including TNF-α, IL-6 and reactive oxygen species which may cause kidney damage and reduced GFR (12). Visfatin also upregulates RAS which plays an important role in regulation of GFR, however, the exact role of visfatin in CKD in obese patients is unclear. Apelin is a new adipokine which act as a vasoactive peptide. It also expressed in the glomeruli and causes inflammation and endothelial dysfunction in CKD (2).

In conclusion, there is a link between adipokines and development of CKD in obesity and this is an emerging field of research which will lead to new strategies for management or treatment of CKD.

## Authors’ contribution

MK is the single author of the manuscript.

## Conflicts of interest

The author declared no competing interests.

## Ethical considerations

Ethical issues (including plagiarism, data fabrication, double publication) have been completely observed by author.

## Funding/Support

No funding from any source is directly associated with this manuscript.
